# Epithelial PCSK6 Promotes Proliferation and Decreases Collagen Deposition by Fibroblasts Potentially via MMP Activation

**DOI:** 10.3390/ijms27115104

**Published:** 2026-06-04

**Authors:** Qian Tian, Annemiek Dijkhuis, Ester B. M. Remmerswaal, Hella Aberson, Bruno Crestani, Tom van der Poll, C. Arnold Spek, Jan Willem Duitman

**Affiliations:** 1 Department of Experimental Immunology, Amsterdam UMC Location University of Amsterdam, 1105 AZ Amsterdam, The Netherlands; a.dijkhuis@amsterdamumc.nl (A.D.); e.b.remmerswaal@amsterdamumc.nl (E.B.M.R.); 2 Center for Infection and Molecular Medicine, Amsterdam UMC Location University of Amsterdam, 1105 AZ Amsterdam, The Netherlands; h.l.aberson@amsterdamumc.nl (H.A.); t.vanderpoll@amsterdamumc.nl (T.v.d.P.); c.a.spek@amsterdamumc.nl (C.A.S.); 3 Service de Pneumologie, Allergologie et Transplantation, Hôpital Bichat Claude Bernard, AP-HP, 75018 Paris, France; bruno.crestani@aphp.fr; 4 Division of Infectious Diseases, Amsterdam UMC Location University of Amsterdam, 1105 AZ Amsterdam, The Netherlands; 5 Laboratory of Experimental Oncology and Radiobiology, Amsterdam UMC Location University of Amsterdam, 1105 AZ Amsterdam, The Netherlands; 6 Department of Pulmonary Medicine, Amsterdam UMC Location University of Amsterdam, 1105 AZ Amsterdam, The Netherlands; 7 Amsterdam Institute for Immunology & Infectious Disease, Amsterdam UMC Location University of Amsterdam, 1105 AZ Amsterdam, The Netherlands

**Keywords:** idiopathic pulmonary fibrosis, matrix metalloproteinases, lung fibroblasts, alveolar epithelial cells, proprotein convertase subtilisin/kexin type 6

## Abstract

A recent genome-wide association study showed that the protease proprotein convertase subtilisin/kexin type 6 (PCSK6) is highly expressed in idiopathic pulmonary fibrosis (IPF) lung parenchyma and that its expression is associated with disease progression and worse survival. However, whether PCSK6 plays a role in IPF pathophysiology remains elusive. This study aimed to determine whether PCSK6 contributes to IPF pathophysiology, specifically in the cross-talk between epithelial cells and fibroblasts. A549 epithelial cells were transduced with a PCSK6-GFP or control-mCherry vector. Both proliferation (crystal violet) and cell competition assays showed that PCSK6 promoted cell proliferation. Western blot and PCSK-specific fluorogenic substrate assays showed that A549-PCSK6 conditioned medium (CM) had higher PCSK6 enzyme activity compared to mCherry control A549 CM. Human lung fibroblasts stimulated with PCSK6-CM significantly decreased collagen I protein levels as compared to fibroblasts stimulated with control A549-CM. Matrix-metalloproteinase (MMP) specific fluorogenic substrate assays subsequently showed that A549-PCSK6 CM contained higher MMP activity and that PCSK6 inhibition reduced MMP activity in A549-PCSK6 CM, suggesting that PCSK6 plays a role in the activation of MMPs that may degrade collagen type I. In conclusion, epithelial PCSK6 promotes cell proliferation and decreases collagen deposition by fibroblasts, potentially via MMP activation. These in vitro data suggest that PCSK6 could play a dual role in IPF progression and its actual role in IPF should consequently be elucidated using in vivo/ex vivo models.

## 1. Introduction

Idiopathic pulmonary fibrosis (IPF) is a progressive interstitial lung disease marked by excessive extracellular matrix (ECM) accumulation, resulting in severe impairment of lung function [1]. Although two drugs are currently approved for IPF treatment [2,3], they neither reverse fibrosis progression nor substantially improve long-term survival. Therefore, unraveling the molecular mechanisms driving fibrosis remains critical to develop effective novel therapies.

Previous work from our group identified protease-activated receptor-1 (PAR-1) as a pivotal player in pulmonary fibrosis [4,5]. PAR-1 is a seven-transmembrane G protein-coupled receptor activated by proteolytic cleavage, initiating intracellular signaling cascades that regulate diverse pathological processes [6,7]. We demonstrated that PAR-1 activation induces extracellular signal-regulated kinase (ERK) phosphorylation in fibroblasts, promoting collagen production and α-smooth muscle actin (α-SMA) expression. Conversely, inhibition of PAR-1 using the antagonist P1pal-12 reduced fibroblast differentiation, proliferation, and migration, and significantly attenuated bleomycin-induced lung fibrosis in mice [4].

Recently, a large-scale, international two-stage genome-wide association study (GWAS) uncovered four novel gene variants linked to IPF survival, with Proprotein Convertase Subtilisin/Kexin type 6 (PCSK6) emerging as a key candidate exhibiting genome-wide significance. Elevated *PCSK6* expression in peripheral blood, increased plasma PCSK6 protein levels, and heightened PCSK6 staining in IPF lung tissue all correlated with worse transplantation-free survival [8]. PCSK6, also known as paired basic amino-acid-cleaving enzyme 4 (PACE4) or subtilisin-like proprotein convertase 4 (SPC4), is a member of the proprotein convertase family involved in the proteolytic maturation of numerous substrates, including matrix metalloproteinases (MMPs), pro-transforming growth factor (TGF)-β, pro-Insulin-like Growth Factor 2 (IGF-2) and various serine proteases [9,10]. The *PCSK6* gene, located on chromosome 15, is widely expressed in human organs, such as the lung, liver, pancreas, kidney and brain [11,12]. Notably, prior studies demonstrated that PCSK6 can cleave PAR-1 [13,14], based on which we previously hypothesized that PCSK6 may be the endogenous PAR-1 agonist driving pulmonary fibrosis [15].

Taken together, it is tempting to speculate that PCSK6 plays a key role in IPF pathogenesis. However, it remains to be determined whether PCSK6 indeed plays a direct role in driving disease progression. In this study, we aimed to clarify the potential role of PCSK6 in IPF. Given that GWAS data showed increased PCSK6 levels in IPF lung parenchyma, particularly within ciliated and alveolar epithelial cells, compared to controls [8], we generated PCSK6-overexpressing A549 alveolar epithelial cells. Interestingly, these PCSK6 overexpressing epithelial cells not only showed enhanced proliferation but also increased extracellular MMP activity that may subsequently reduce collagen deposition of fibroblasts.

## 2. Results

### 2.1. PCSK6 Overexpression Induces Proliferation of A549 Epithelial Cells Independent of PAR-1

**Figure 1:**
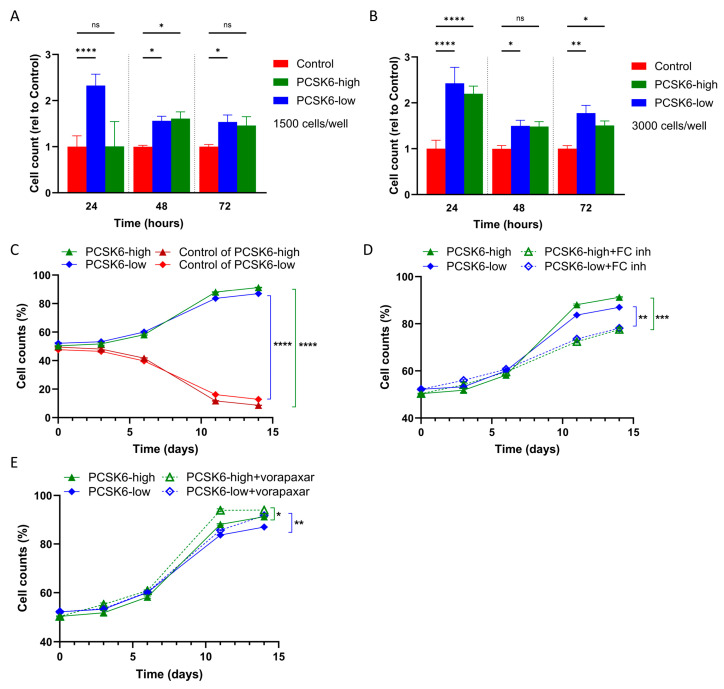
PCSK6 overexpression promotes epithelial (A549) cell proliferation. (**A**,**B**) Crystal violet proliferation assay showing relative cell concentrations at 24, 48, and 72 h of culture started with 1500 cells/well (**A**) or 3000 cells/well (**B**) (*n* = 6). A representative graph of three independent experiments performed with six replicates per group is shown. (**C**) Flow cytometry analysis showing the percentage of mCherry-control (red line), PCSK6-high (green line) and PCSK6-low (blue line) fluorescent cells in co-culture over time (*n* = 3). (**D**,**E**) Flow cytometry analysis showing the percentage (%) of mCherry-control cells in co-culture with PCSK6-high or PCSK6-low A549 cells with or without 50 µM furin convertase inhibitor (FC inh, (**D**)) or 1 µM vorapaxar (**E**). (*n* = 3). A representative graph of two independent experiments performed with three replicates per group is shown. Results are expressed as mean ± SEM. Statistical significance was tested using one-way (**A**,**B**) or mixed-effects analysis with Tukey’s post hoc test for multiple comparisons (**C**–**E**), and significance is shown relative to the indicated groups. *p*-values: * <0.05, ** <0.01, *** <0.001 and **** <0.0001.

Given the elevated levels of PCSK6 in IPF lung parenchyma, mainly in epithelial cells [8], PCSK6 overexpression was induced in A549 lung epithelial cells and proliferation was assessed. As shown in Figure 1A,B, PCSK6 overexpression induced a significant increase in A549 cell proliferation at 24, 48, and 72 h in both PCSK6-high and PCSK6-low A549 cells compared to mCherry-control cells (Figure 1A,B; starting with 1500 and 3000 cells/well, respectively).

Furthermore, PCSK6-overexpressing cells showed a significant growth advantage compared with mCherry controls when cultured together (Figure 1C), suggesting that the effect on proliferation is cell-intrinsic and not dependent on paracrine signaling. Indeed, the proportion of PCSK6-high cells increased markedly from 50.37 ± 0.22% on day 0 to 91.25 ± 0.26% on day 14. Similarly, the PCSK6-low proportion increased from 52.23 ± 0.52% to 87.05 ± 0.10% over the same time period (Figure 1C). Treatment with a furin convertase inhibitor known to inhibit PCSK6 activity significantly attenuated the proliferation advantage of PCSK6-overexpressing cells in both PCSK6-high and PCSK6-low co-cultures (Figure 1D), showing that the proliferation advantage is indeed dependent on PCSK6. In order to test whether the increased proliferation was dependent on PAR-1 activation by PCSK6, cells were treated with the PAR-1 inhibitor vorapaxar. As shown in Figure 1E, vorapaxar did not inhibit the growth advantage of PCSK6-overexpressing cells, indicating that PCSK6-mediated proliferation is independent of PAR-1 signaling.

### 2.2. PCSK6 Activity in CM Can Be Inhibited by the Furin Convertase Inhibitor Chloromethylketone

**Figure 2:**
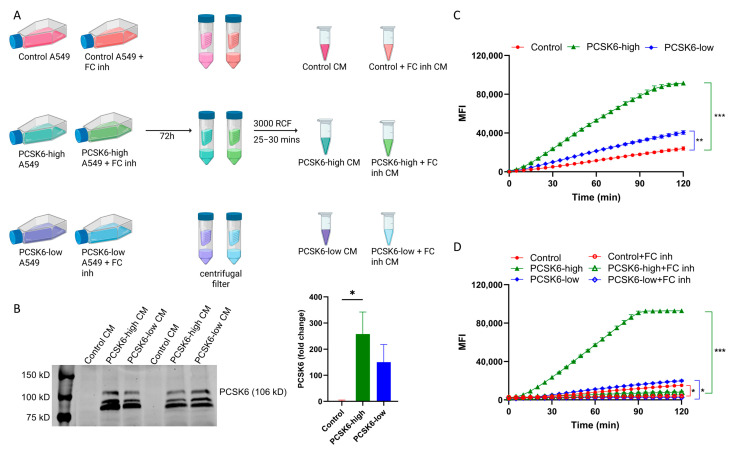
Enzymatic activity of PCSK6 is increased in CM of PCSK6-high and PCSK6-low A549 cells. (**A**) Schematic illustration of conditioned medium (CM) collection of different A549 cell lineages with or without 50 µM furin convertase inhibitor (FC inh). (**B**) Representative Western blot image of PCSK6 levels in PCSK6-high, PCSK6-low and mCherry-control CM demonstrating increased levels of secreted PCSK6 in PCSK6-high and PCSK6-low CM. Predicted molecular weight of PCSK6 is 106 kD. The three lanes on the right are a duplicate of the three lanes on the left. Quantification of the densitometry is shown relative to Control CM. (**C**,**D**) Proteolytic activity of PCSK6 in mCherry-control (red line), PCSK6-high (green line) and PCSK6-low (blue line) A549 cell CM collected in the absence (**C**) or presence (**D**) of 50 µM furin convertase inhibitor (FC inh) using a specific fluorescent furin convertase substrate (Boc-Arg-Val-Arg-Arg-AMC; *n* = 3 per group). Fluorescence was recorded at excitation 360/40 nm and emission 485/20 nm and normalized to the baseline fluorescence of the substrate peptide in medium alone. MFI indicates mean channel fluorescence minus background. Results are expressed as mean ± SEM. Statistical significance was tested using mixed-effects analysis with Tukey’s post hoc test for multiple comparisons, and significance is shown relative to the indicated groups. *p*-values: * <0.05, ** <0.01, *** <0.001.

To determine the effect of secreted PCSK6 on fibroblasts, mCherry-control, PCSK6-high and PCSK6-low conditioned medium (CM) from cells pretreated with or without furin convertase inhibitor were collected (Figure 2A). Both Western blot and a PCSK6-specific fluorogenic substrate assay were performed to confirm the presence and activity of PCSK6 in CM (Figure 2B,C). Western blot analysis demonstrated elevated levels of PCSK6 protein in both PCSK6-high and PCSK6-low CM compared to mCherry-control CM (Figure 2B). Moreover, CM from PCSK6-high and PCSK6-low A549 cells showed significant PCSK6 activity compared to CM from mCherry-control cells (Figure 2C). Importantly, CM of furin convertase inhibitor-treated PCSK6-high, PCSK6-low A549 cells showed a significant reduction in PCSK6 activity (Figure 2D), suggesting that PCSK6 in the CM is enzymatically active and can be effectively inhibited by furin convertase inhibitor.

### 2.3. PCSK6 CM Reduces Collagen I Deposition of Fibroblasts Derived from Healthy and IPF Patients

**Figure 3:**
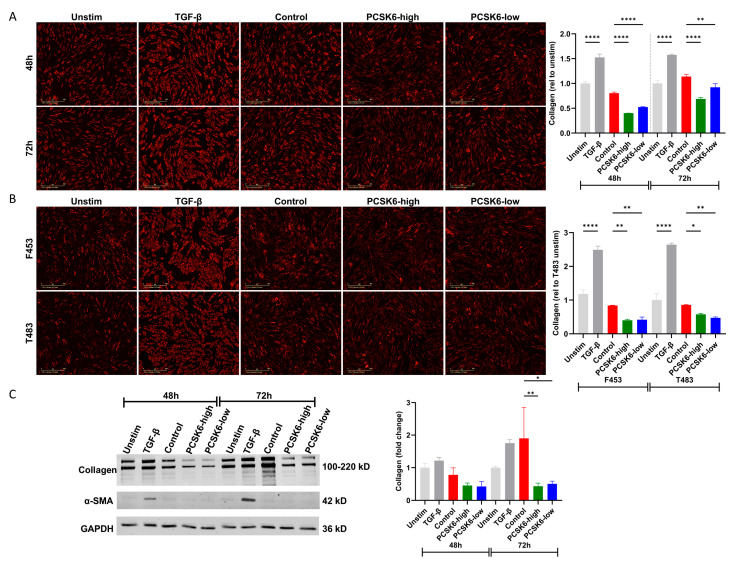
Epithelial PCSK6 reduces collagen deposition by fibroblasts from both healthy individuals and IPF patients. (**A**) Representative images (10× magnification) of immunofluorescence staining for collagen type I in NHLFs 48 and 72 h after stimulation with mCherry-control, PCSK6-high and PCSK6-low CM. Quantification of the fluorescent signal corrected for confluence is shown relative to unstimulated NHLFs. TGF-β stimulation served as a positive control (*n* = 4). Scale bar = 400 μm. (**B**) Representative images of immunofluorescence staining for collagen type I in fibroblasts derived from an IPF patient (F453) and a healthy control (T483) 48 h after stimulation with mCherry-control, PCSK6-high and PCSK6-low CM. Quantification of the fluorescent signal corrected for confluence is shown relative to unstimulated fibroblasts. TGF-β stimulation served as a positive control (*n* = 3). Scale bar = 400 µm. (**C**) Representative Western blot image of collagen type I, αSMA and GAPDH in NHLF cell lysates after 48 and 72 h of stimulation with conditioned media. Quantification of collagen type I Western blot is shown relative to unstimulated cells and corrected for GAPDH levels, which served as a loading control. Results were expressed as mean ± SEM. Statistical significance was tested using one-way ANOVA. *p*-values: * <0.05, ** <0.01 and **** <0.0001.

**Figure 4:**
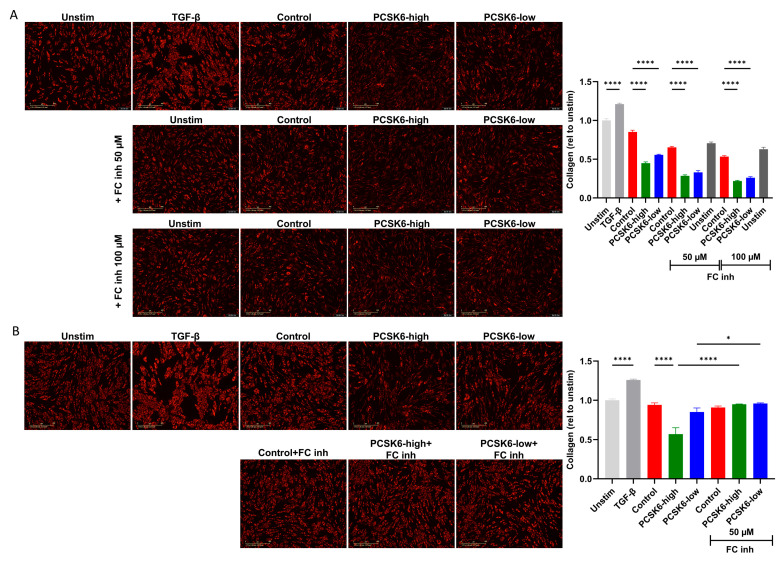
Epithelial PCSK6 reduces collagen deposition by fibroblasts independent of PCSK6 transfer. (**A**) Representative images (10× magnification) of immunofluorescence staining for collagen type I in NHLFs 48 h after stimulation with mCherry-control, PCSK6-high and PCSK6-low CM in the presence or absence of 50 µM or 100 µM furin convertase inhibitor (FC inh). The FC inhibitor was added to the NHLFs 1 h prior to CM stimulation. Quantification of the fluorescent signal corrected for confluence is shown relative to unstimulated NHLFs. TGF-β stimulation served as a positive control (*n* = 3). Scale bar = 400 µm. (**B**) Representative images (10× magnification) of immunofluorescence staining for collagen type I in NHLFs 48 h after stimulation with mCherry-control, PCSK6-high and PCSK6-low CM collected in the presence or absence of 50 µM furin convertase inhibitor (FC inh). A549 cells were treated with the FC inhibitor for 72 h before CM collection. Quantification of the fluorescent signal corrected for confluence is shown relative to unstimulated NHLFs. Scale bar = 400 μm. TGF-β stimulation served as a positive control (*n* = 3). Results are expressed as mean ± SEM. Statistical significance was tested using one-way ANOVA. *p*-values: * <0.05, **** <0.0001.

To investigate the effect of PCSK6 activity on human lung fibroblasts, the key effector cells in pulmonary fibrosis, normal human lung fibroblast (NHLF), IPF patient-derived fibroblasts (F453) and healthy control fibroblasts (T483) were stimulated with CM as collected. As shown in Figure 3A (immunofluorescence) and C (Western blot), a significant reduction in collagen levels of NHLFs treated with PCSK6-high and PCSK6-low CM was observed compared to the mCherry-control after 48 and 72 h of stimulation. Interestingly, α-SMA and fibronectin levels were not affected by CM stimulation (Figure S2). In line, a significant reduction in collagen levels was observed in primary lung fibroblasts derived from an IPF patient and a control individual incubated in PCSK6-containing CM but not control CM (Figure 3B). Rather unexpectedly, pre-stimulation of fibroblasts with the furin convertase inhibitor did not reverse the collagen reduction induced by PCSK6-high and PCSK6-low CM (Figure 4A), suggesting that the transferred PCSK6-mediated reduction in collagen was already derived in the A549 cells. Indeed, CM from PCSK6-high and PCSK6-low A549 cells treated with the furin convertase inhibitor 72 h prior to CM collection failed to reduce collagen deposition of fibroblasts (Figure 4B), suggesting that the factors responsible for the collagen reduction are produced in a PCSK6-dependent manner in the A549 culture.

### 2.4. PCSK6 Reduces Collagen Deposition Potentially via MMP Activation

To determine whether the observed decrease in collagen after incubation of fibroblasts in PCSK6 CM was due to degradation rather than reduced production, the mRNA levels of collagen I in fibroblasts were measured. As shown in Figure S3A, *COL1A1* expression was not significantly reduced in fibroblasts treated with either PCSK6-high or PCSK6-low CM compared to mCherry-control CM, suggesting that the reduced collagen deposition observed in fibroblasts exposed to PCSK6-CM was likely due to increased collagen degradation rather than decreased de novo synthesis.

**Figure 5:**
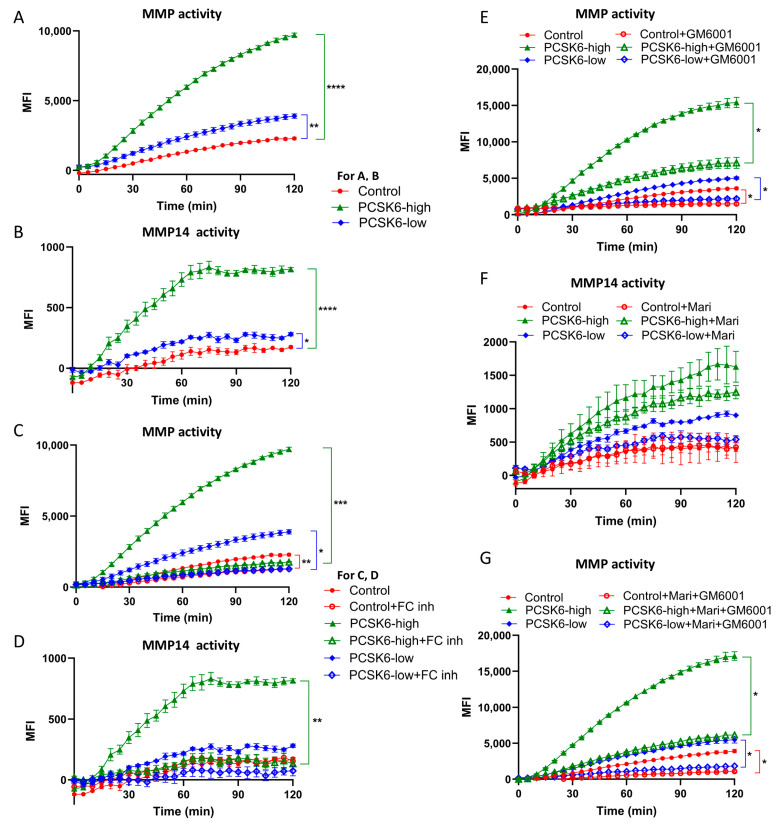
PCSK6 promotes MMP activation. (**A**) Proteolytic activity of CM from mCherry-control, PCSK6-high and PCSK6-low A549 cells on the fluorogenic MMP substrate peptide {Mca}-Lys-Pro-Leu-Gly-Leu-{Dap(Dnp)}-Ala-Arg-NH2 (*n* = 3). (**B**) Proteolytic activity of CM from mCherry-control, PCSK6-high and PCSK6-low A549 cells on the fluorogenic MMP14 substrate peptide MCA-PLA-C(OMeBz)-WAR(Dpa)-NH_2_ (*n* = 3). (**C**) Proteolytic activity of CM from mCherry-control, PCSK6-high and PCSK6-low A549 cells collected in the presence or absence of 50 µM furin convertase inhibitor (FC inh) on the fluorogenic MMP substrate peptide (*n* = 3). (**D**) Proteolytic activity of CM from mCherry-control, PCSK6-high and PCSK6-low A549 cells collected in the presence or absence of 50 µM furin convertase inhibitor (FC inh) on the fluorogenic MMP14 substrate peptide (*n* = 3). (**E**–**G**) Proteolytic activity of CM from mCherry-control, PCSK6-high and PCSK6-low A549 cells in the presence or absence of 500 µM broad-spectrum MMP inhibitor GM6001 (**E**), 50 µM Marimastat (**F**) or the combination of GM6001 and Marimastat (**G**). Fluorescence was measured at excitation 320/20 nm, emission 360/40 nm and normalized to the baseline fluorescence of the substrate peptide in the medium alone. MFI indicates mean channel fluorescence minus background. Data are expressed as mean ± SEM. Statistical significance was tested using mixed-effects analysis with Tukey’s post hoc test for multiple comparisons, and significance is shown relative to the indicated groups. *p*-values: * <0.05, ** <0.01, *** <0.001 and **** <0.0001.

MMPs are key enzymes involved in extracellular matrix remodeling, particularly collagen degradation [16,17,18]. To determine whether PCSK6 overexpression in A549 cells led to MMP activation, a general fluorogenic MMP substrate, cleaved by a broad spectrum of MMPs, was used. Incubation of this general substrate with recombinant MMP-1 produced a strong fluorescence signal, suggesting efficient cleavage (Figure S3B). Conditioned media from mCherry-control, PCSK6-high, and PCSK6-low A549 cells all activated the MMP substrate, but MMP activity was significantly higher in PCSK6-high and PCSK6-low CM (Figure 5A). PCSK6 activity (shown in Figure 2B) and MMP activity as measured by substrate cleavage in both PCSK6-high and PCSK6-low CM were strongly correlated (Figure S3C,D for PCSK6-high: r = 0.9983, *p* < 0.0001 and PCSK6-low: r = 0.9963, *p* < 0.0001, respectively), suggesting a functional link between PCSK6 and MMP activity. Given prior studies showing activation of MT1-MMP/MMP14 by PCSK6 [19,20], a fluorogenic substrate assay specific for MMP14 activity was employed next. PCSK6-high and PCSK6-low CM showed significantly higher MMP14 activity compared to mCherry-control (Figure 5B), although the total signal was lower compared to the general fluorogenic MMP substrate, suggesting less efficient cleavage. Of note, this activity was again significantly correlated with the PCSK6 activity (Figure S3C,D for PCSK6-high: r = 0.9388 *p* < 0.0001 and PCSK6-low: r = 0.9312, *p* < 0.0001, respectively).

In order to validate the association between PCSK6 expression and MMP activity, MMP activity was assessed in CM of A549 cells treated with the furin convertase inhibitor. As shown in Figure 5C,D, MMP activity was significantly lower in the CM of A549 cells pretreated with furin convertase inhibitor before CM collection (Figure 5C,D; general MMP and MMP14 specific substrate, respectively). Furthermore, we validated increased MMP activity by treating the CM with the broad-spectrum MMP inhibitor GM6001 (500 µM), the broad-spectrum MMP inhibitor Marimastat (50 µM) or a combination of both. As shown in Figure 5E,G, MMP inhibition using GM6001 or the combination of GM6001 and Marimastat significantly inhibited MMP substrate cleavage by PCSK6-CM. Treatment with Marimastat alone also inhibited MMP activity in PCSK6-CM, but this did not reach statistical significance (Figure 5F). Taken together, these results show that PCSK6 overexpression leads to increased MMP activity in CM of PCSK6-high and PCSK6-low A549 cells.

## 3. Discussion

Previously, Oldham and colleagues showed that PCSK6 levels are increased in the lung parenchyma of IPF patients, predominantly in epithelial cells [8]. After the identification of a genome-wide significant *PCSK6* variant in a large staged genome-wide association study, PCSK6 protein levels were found to be significantly associated with pulmonary fibrosis progression and transplant-free survival. Here, we aimed to investigate the potential role of PCSK6 in pulmonary fibrosis, specifically in the cross-talk between epithelial cells and fibroblasts. We show that overexpression of PCSK6 in A549 epithelial cells increased proliferation, providing these cells with a growth advantage over controls (Figure 1). PCSK6 overexpression also increased MMP activity in CM from A549 cells (Figure 5), and transfer of this CM to lung fibroblasts significantly reduced collagen levels (Figure 3 and Figure 4), while differentiation and fibronectin levels remained unaffected (Figure S2).

Proliferation of PCSK6 overexpressing A549 cells was increased compared to control cells (Figure 1), consistent with previous reports implicating PCSK6 in cell proliferation. PCSK6 is overexpressed in pancreatic cancer cells and is significantly associated with poor survival; functional assays demonstrated that PCSK6 inactivation reduces proliferation through G0/G1 cell cycle arrest in vitro and decreases tumor burden and metastasis in vivo [21]. Similarly, PCSK6 expression in fibroblast-like synoviocytes from rheumatoid arthritis patients promotes proliferation [22]. In pulmonary fibrosis, epithelial proliferation and hyperplasia appear to promote a profibrotic environment, whereas inhibition of epithelial proliferation alleviates fibrosis [23,24]. Moreover, the proliferation of a specific subset of aberrant alveolar epithelial cells contributes to fibrosis development and progression [25]. Therefore, increased proliferation driven by PCSK6 expression may likely be detrimental in the context of pulmonary fibrosis. Together with the observed reduced collagen deposition induced by PCSK6 (Figure 3) this suggests that PCSK6 may have a dual role in the development of pulmonary fibrosis.

Interestingly, PCSK6 overexpression led to increased MMP activity in CM, as evidenced by enhanced cleavage of MMP-specific substrates (Figure 5A,B). Pre-incubation of these substrates with MMP inhibitors reduced cleavage, confirming MMP involvement. We attempted to identify the specific MMP(s) activated by PCSK6 that were responsible for the observed decrease in collagen type I deposition by lung fibroblasts. However, the substrates used are not specific to individual MMPs, which obscured our attempts. The FS 6 (CAS 720710 69 0) fluorogenic substrate can be cleaved by a broad range of MMPs, including the collagenases MMP1, MMP8, and MMP13 [26]. The MMP14 fluorogenic substrate is not only cleaved by MMP14 but also, rather efficiently, by MMP11, although MMP11 is not considered a collagenase due to its lack of activity against native type I collagen [27]. Our results show increased proteolytic activity for both substrates, but the stronger signal obtained with the general MMP substrate (Figure 5A) suggests that multiple MMPs are active in the conditioned medium. Likewise, the MMP inhibitors used are not specific to single MMPs, and the incomplete inhibition observed in Figure 5E–G further supports the involvement of multiple MMPs. Several studies have reported links between PCSK6 expression and MMP activation. Rykaczewska et al. found that PCSK6 colocalizes and activates MMP14/MMP2 in smooth muscle α-actin+ cells, and that PCSK6-deficient mice show suppressed extracellular matrix-remodeling enzyme activity [19]. Another study demonstrated that MMP14 and MMP15 activity is increased in transgenic PCSK6 overexpressing keratinocytes, resulting in collagenase activation and collagen degradation [20]. Additionally, PCSK6 and MMP9 activity were associated in U937 cells and HUVECs treated with oxysterols and the aldehyde 4-hydroxy-2-nonenal (HNE), major pro-atherogenic components of oxidized LDLs. Downregulation of PCSK6 by siRNA significantly blocked MMP9 activity induced by oxysterols and HNE, suggesting a functional link between PCSK6 and MMP9 [28]. Conversely, Kuhn and colleagues [29] reported that PCSK6 acts as a profibrotic protein by enhancing collagen production in fibroblasts during cardiac remodeling after myocardial infarction, indicating that the role of PCSK6 may be context-dependent.

We previously hypothesized that PCSK6 could be the endogenous PAR-1 agonist driving pulmonary fibrosis [15]. PCSK6 is indeed a potent PAR-1 agonist [13,14], and proteolytic activation of PAR-1 in lung fibroblasts contributes to proliferation and fibrotic responses [4], suggesting that PCSK6 could drive fibrosis in a PAR-1 dependent manner. However, inhibition of PAR-1–dependent pathways did not affect the PCSK6-dependent increase in A549 proliferation (Figure 1) or the observed decreased collagen deposition of fibroblasts exposed to PCSK6 high or PCSK6 low conditioned medium (Figure S4). Of note, the observed PCSK6-dependent decrease in collagen deposition (Figure 3) is opposite to the previously reported increased fibrotic responses induced by PAR-1 activation [4]. We therefore conclude that PCSK6 is not the endogenous PAR-1 agonist driving cell proliferation and fibrotic responses of fibroblasts. The identity of the PAR-1 agonist responsible for fibrosis progression thus remains unknown.

This study has several limitations. First, the effects of PCSK6 on fibrotic responses were examined only in vitro, which does not fully capture the complexity of the in vivo environment. Conditioned medium transfer from A549 epithelial cells to lung fibroblasts allowed us to study the interplay between these cell types and provided insight into the mechanisms by which PCSK6 influences pulmonary fibrosis. Moreover, A549 cells, although widely used as a model for respiratory epithelium, are cancer-derived cells bearing alveolar type II epithelial cell features, but do not fully reflect primary alveolar epithelial cells or epithelial cells from the lungs of patients with IPF. Manipulation of PCSK6 activity in vivo or in ex vivo systems such as precision-cut lung slices would be required to determine the net effect of PCSK6 on fibrosis progression, but these experiments were beyond the scope of this study. Second, the MMP substrates and inhibitors used lack specificity for individual MMPs, complicating identification of the MMPs responsible for the observed effects. Additionally, the relationship between MMP activity and reduced collagen deposition remains indirect. To address this issue, several experiments were performed using MMP inhibitors to reverse the decrease in collagen induced by PCSK6-CM (Figure S5). GM6001 and marimastat alone did not reverse the collagen decrease significantly, suggesting that multiple MMPs are responsible for the observed effect, which would be in line with the substrate experiments showing multiple MMP activities in PCSK6-CM. Unfortunately however, combining GM6001 and marimastat induced toxicity of the NHLFs, making it impossible to draw conclusions on a direct relationship between MMP activity and reduced collagen deposition. Despite these limitations, our results suggest that PCSK6 activates multiple MMPs rather than a single one, and that targeting PCSK6 could increase collagen deposition, which would likely be harmful in pulmonary fibrosis.

## 4. Materials and Methods

### 4.1. Cells and Culture Conditions

A549 cells (obtained from ATCC, Manassas, VA, USA, cat.nr.: CCL-185), HEK293T cells (obtained from ATCC, cat. nr. CRL-3216), normal human lung fibroblasts (NHLF, obtained from Lonza Bioscience, Basel, Switzerland, cat. nr. CC-2512) and primary control (T483) and IPF (F453) fibroblasts (kindly provided by prof. dr. Bruno Crestani) were maintained in DMEM (11960-044, Gibco, Grand Island, NY, USA) supplemented with 10% (*v*/*v*) fetal bovine serum (FBS, F9665, Sigma-Aldrich, St. Louis, MO, USA), 2 mM L-glutamine (25030-024, Gibco) and 1% (*v*/*v*) penicillin/streptomycin (15140-122, Gibco) at subconfluency in a tissue culture incubator at 37 °C in a humidified atmosphere containing 5% CO_2_. F453 and T483 fibroblasts were isolated from IPF and control lung tissue samples, as previously described [30]. Donors of the primary cells provided informed consent and the collection of primary cells was approved by the institutional ethics committee (Comité d’éthique du CEERB Paris Nord, biobank registration number DC 2009-940).

### 4.2. Production of mCherry-Control and PCSK6-GFP Overexpressing A549 Cells

Control and PCSK6 overexpressing cells were prepared using a pcDNA3.1+ vector containing a mCherry coding insert (control-mCherry, VB010000-9390NKA) and a pcDNA3.1+ vector containing a PCSK6-GFP coding insert (PCSK6-GFP, VB230310-1234GEU), which were obtained from Vector Builder (Neu-Isenburg, Germany). *Escherichia coli* bearing the plasmids were amplified overnight in Lysogeny-Broth (LB) medium and plasmid DNA was isolated using a Midiprep kit (740410.50, Macherey-Nagel, Düren, Germany) following the manufacturer’s instructions. For the production of PCSK6-GFP or control-mCherry bearing lentiviruses, a third-generation lentiviral system in HEK293T cells was used as described previously [31]. In short, HEK293T cells were transfected using Lipofectamine 2000 transfection reagent (11668-019, Invitrogen, Carlsbad, CA, USA). The viral supernatant was collected at 48 h after transfection and used to transduce A549 cells. Two days after viral transduction, puromycin (100552, ICN Biochemicals, Aurora, OH, USA) was added to select for successfully transduced cells.

### 4.3. Cell Sorting for Single Cell Clones and Generating Pools

GFP- and mCherry-positive single cells were sorted using a Sony SH800 cell sorter (Sony Biotechnology, San Jose, CA, USA) equipped with a 130 µm nozzle. Sorted single cells were seeded into 96-well plates and expanded. Once confluent, cells were transferred to 24-well plates and subsequently to culture flasks for further expansion. PCSK6 levels were assessed by Western blot and fluorescence detection (GFP or mCherry) using an IncuCyte imaging system (Sartorius, Göttingen, Germany). As shown in Figure S1, single cell clones expressed mCherry (control) or GFP. Based on PCSK6 levels (Figure S1C), two GFP-positive clones (clone 4 and 5) were pooled to generate high PCSK6-expressing cells (PCSK6-high pool), while two clones (clone 1 and 2) were combined to generate intermediate PCSK6-expressing cells (PCSK6-low pool). Four mCherry-positive clones (clone 2, 3, 4, and 5) were pooled to generate the mCherry-control pool. These pools were used for further mechanistic studies.

### 4.4. Western Blot Analysis

Cells were lysed using RIPA lysis buffer supplemented with protease inhibitor (04693124001, Roche Diagnostic GmbH, Mannheim, Germany) and phosphatase inhibitor (04906837001, Roche). Protein concentrations were determined using the BCA Protein Assay Kit (23227, Thermo Scientific, Rockford, IL, USA). Equal amounts of proteins were mixed with sample buffer and DTT and denatured by incubation at 95 °C for 5 min. Proteins were separated on 10% SDS-PAGE gels using Mini-PROTEAN equipment (Bio-Rad Laboratories, Hercules, CA, USA), and subsequently transferred to PVDF membranes (IPFL00010, Millipore, Bedford, MA, USA) at 240 mA for 80 min. After incubating in 2% non-fat milk solution to block nonspecific antigens for 1 h at room temperature, the membrane was incubated overnight at 4 °C with primary antibody diluted in 0.5% non-fat milk solution, including PCSK6 (abx028342, Abbexa, Cambridge, UK), α-tubulin (sc-23948, Santa Cruz Biotechnology, Dallas, TX, USA), type I collagen-UNLB (Col1; 1310-01, Southern Biotech, Birmingham, AL, USA), α-SMA (sc-32251, Santa Cruz) and GAPDH (sc-31915, Santa Cruz). After washing, membranes were incubated with IRDye-conjugated secondary antibodies (LI-COR Biosciences, Lincoln, NE, USA): anti-rabbit (926-68073, 926-32213), anti-goat (926-32214) and/or anti-mouse (926-68072). Imaging and quantification were performed using an Odyssey imaging system (LI-COR).

### 4.5. Proliferation Assay

Cell proliferation was measured using a crystal violet staining assay. A549 mCherry-control, PCSK6-high and PCSK6-low cells were seeded into 96-well plates at densities of 1500 or 3000 cells per well, with six technical replicates per condition. On the following day, the cells were serum-starved in DMEM containing 0.5% FBS. At 24 h, 48 h, and 72 h post-starvation, the medium was removed and the cells were washed with PBS. Cells were fixed and stained with a solution containing 6% glutaraldehyde (CAS 111-30-8, Sigma-Aldrich) and 0.5% crystal violet (CAS 548-62-9, Thermo Scientific) for 30 min. After three washes with water to remove the residual unbound crystal violet, DMSO was added to dissolve the dye. Absorbance was measured at 600 nm using a BioTek Synergy HT microplate reader (BioTek Instruments, Winooski, VT, USA) to quantify the amount of crystal violet that was bound to the cell membrane as a measure of cell numbers.

### 4.6. Cell Competition Assay

Flow cytometry (FACS) was used to assess competitive growth dynamics among A549 mCherry-control, PCSK6-high, and PCSK6-low cell populations. Equal numbers of cells were seeded into 6- or 12-well plates under the following conditions: normal A549, mCherry-control, PCSK6-high, PCSK6-low, 50% mCherry-control + 50% PCSK6-high, 50% mCherry-control + 50% PCSK6-low, 50% mCherry-control + 50% PCSK6-high. In specific experiments, 50 µM of the furin convertase inhibitor chloromethylketone (ALX-260-022, Enzo Life Sciences, Farmingdale, NY, USA) or 1 µM of the PAR-1 inhibitor Vorapaxar (SCH 530348, AdooQ Bioscience, Irvine, CA, USA) was used to inhibit PCSK6 or PAR-1 activity, respectively. Experiments were performed in quadruplicate. Once cells reached confluence, three wells per condition were harvested for FACS analysis using the ECD and FITC channels on a CytoFLEX S cytometer (Beckman Coulter Life Sciences, Brea, CA, USA) to quantify mCherry and GFP-positive cells. The cells in the remaining well were passed to four new wells for continued culture. This process was repeated over a 14-day period, with FACS analysis on days 3, 6, 11 and 14 to monitor population dynamics.

### 4.7. Preparation of Control and PCSK6 CM

Equal numbers of A549 mCherry-control, PCSK6-high, and PCSK6-low cells were seeded into T75 culture flasks (430725U, Corning, Corning, NY, USA). Once cells reached approximately 70–80% confluence, the medium was replaced with DMEM containing 0.5% FBS. After 72 h of incubation, the CM was collected and concentrated using 3 kDa Amicon Ultra-15 centrifugal filters (UFC900324, Merck Millipore, Bedford, MA, USA), following the manufacturer’s instructions. Samples were centrifuged at 3000× *g* for 20–30 min to a final volume of 1.5 mL (appr. 10 times concentrated). The concentrated CM was aliquoted and stored at −80 °C until further use. See also Figure 2A for a schematic representation of the CM collection.

### 4.8. Evaluation of PCSK6 and MMP Enzymic Activity and Validity of Inhibitors by Fluorogenic Furin and MMP Substrate Assay

The enzymatic activity of PCSK6 in concentrated conditioned media was measured using a fluorogenic furin substrate peptide (ALX-260-040, Enzo Life Sciences) [28], with or without the furin convertase inhibitor chloromethylketone (ALX-260-022, Enzo Life Sciences). MMP activity was assessed using a fluorogenic MMP substrate peptide (CAS 720710-69-0, MCE, Monmouth Junction, NJ, USA), in the presence or absence of the broad-spectrum MMP inhibitor GM6001 (sc-203979, Santa Cruz Biotechnology) or Marimastat (CAS 154039-60-8, MCE). MMP14/MT1-MMP and MMP11 activity was evaluated using a specific fluorogenic MMP14 substrate peptide (444258, Millipore Sigma, Burlington, MA, USA). Assays were performed in non-treated black 96-well plates (3915, Costar, Corning, NY, USA). Conditioned media were serially diluted 2-fold in cascade using assay buffer (50 mM Tris, pH 8.0; 500 mM NaCl; 0.1× Triton X-100). Samples were prepared on ice in a final volume of 20 µL per well. Reactions were initiated by adding 20 µL of substrate solution to each well at a final concentration of 160 µM. Fluorescence was measured every 5 min for 2 h at 37 °C using a BioTek plate reader at 360/40 nm and 485/20 nm for excitation and emission, respectively, for the PCSK6 substrate, and 320/20 nm and 360/40 nm for the MMP and MMP14 substrates. Fluorescence values were normalized to baseline readings obtained from substrate incubated with fresh DMEM.

### 4.9. Immunofluorescence Staining

Fibroblasts were seeded at a density of 7000 cells per well in 96-well plates. The following day, cells were washed with PBS and incubated in serum-free medium for 6 h. Subsequently, cells were stimulated with conditioned media derived from mCherry-control, PCSK6-high and PCSK6-low cells in a 1:3 or 1:7 (*v*/*v*) ratio. After 48 or 72 h of stimulation, the medium was removed, and the cells were fixed with ice cold 100% methanol for 2 min. Next, cells were blocked with 3% BSA for 1 h at room temperature and incubated with primary antibodies overnight at 4 °C. Primary antibodies used were: α-SMA (sc-32251, Santa Cruz), Col1 (1310-01, Southern Biotech) and Fibronectin (FN; sc-8422, Santa Cruz). The next day, cells were incubated with the appropriate secondary fluorescent antibodies: Alexa Fluor™ 647 (A21447, Thermo Fisher Scientific) or Alexa Fluor™ 488 (A11029, Thermo Fisher Scientific) for 1 h at room temperature in the dark. After two PBS washes, cells were maintained in PBS and imaged using an IncuCyte analysis system (Sartorius).

### 4.10. Statistical Analysis

All data are expressed as mean ± standard error of the mean (SEM). Statistical significance was determined using one-way or mixed effects analysis followed by Tukey’s post hoc test for multiple comparisons. Correlation analysis was computed with Pearson correlation. A *p*-value < 0.05 was considered statistically significant. All statistical analyses were performed using GraphPad Prism version 10.2.0.

## 5. Conclusions

Overall, we conclude that PCSK6 promotes A549 epithelial cell proliferation, indicating that PCSK6 may aggravate aberrant epithelial cell progression during IPF development. On the contrary, we show that PCSK6 increases activation of MMPs secreted by A549 epithelial cells, which may subsequently reduce collagen deposition by fibroblasts. PCSK6 expression may therefore play a dual role in IPF, but its actual role in IPF still remains ambiguous and should be elucidated using in vivo/ex vivo models.

## Data Availability

The original contributions presented in this study are included in the article/Supplementary Materials. Further inquiries can be directed to the corresponding authors.

## Supplementary Materials

The supporting information can be downloaded at https://www.mdpi.com/article/10.3390/ijms27115104/s1.

## Abbreviations

The following abbreviations are used in this manuscript:

PCSK6	Proprotein convertase subtilisin/kexin type 6
IPF	Idiopathic pulmonary fibrosis
PAR-1	Protease-activated receptor-1
ECM	Excessive extracellular matrix
ERK	Extracellular signal-regulated kinase
α-SMA	α-smooth muscle actin
PACE4	Paired basic amino-acid-cleaving enzyme 4
SPC4	Subtilisin-like proprotein convertase 4
MMPs	Matrix metalloproteinases
TGF-β	Transforming growth factor-β
IGF-2	Insulin-like Growth Factor 2
HNE	4-hydroxy-2-nonenal
NHLF	Normal human lung fibroblast
FACS	Flow cytometry
CM	Conditioned medium
SEM	Standard error of the mean
