# Supplementary material for: Epithelial PCSK6 Promotes Proliferation and Decreases Collagen Deposition by Fibroblasts Potentially via MMP Activation
Source: Int J Mol Sci. 2026 Jun 4;27(11):5104. doi: 10.3390/ijms27115104 (PMC13258461; doi:10.3390/ijms27115104)
Supplement: Supplementary file 1 [file ijms-27-05104-s001.zip › ijms-4327757-supplementary.pdf]

Epithelial PCSK6 promotes proliferation and decreases collagen deposition by fibroblasts potentially via MMP activation

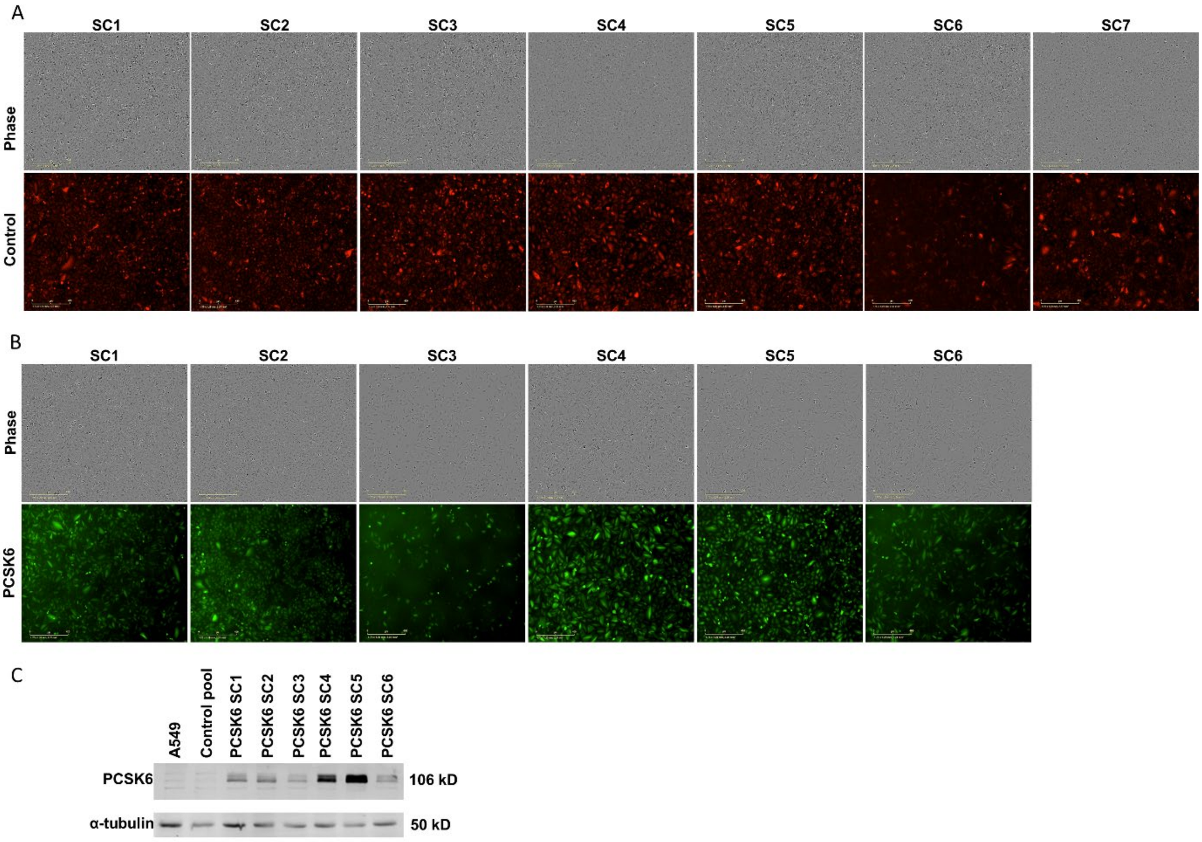

Figure S1. Selection of single cell clones overexpressing PCSK6-GFP and mCherry-control. (A-B) Brightfield (upper) and fluorescent (lower) images of (A) mCherry-control single cell clones and (B) PCSK6-GFP single cell clones. (C) Western Blot image of PCSK6 and α-tubulin in A549, mCherry-control pool and PCSK6-GFP single cell clones.

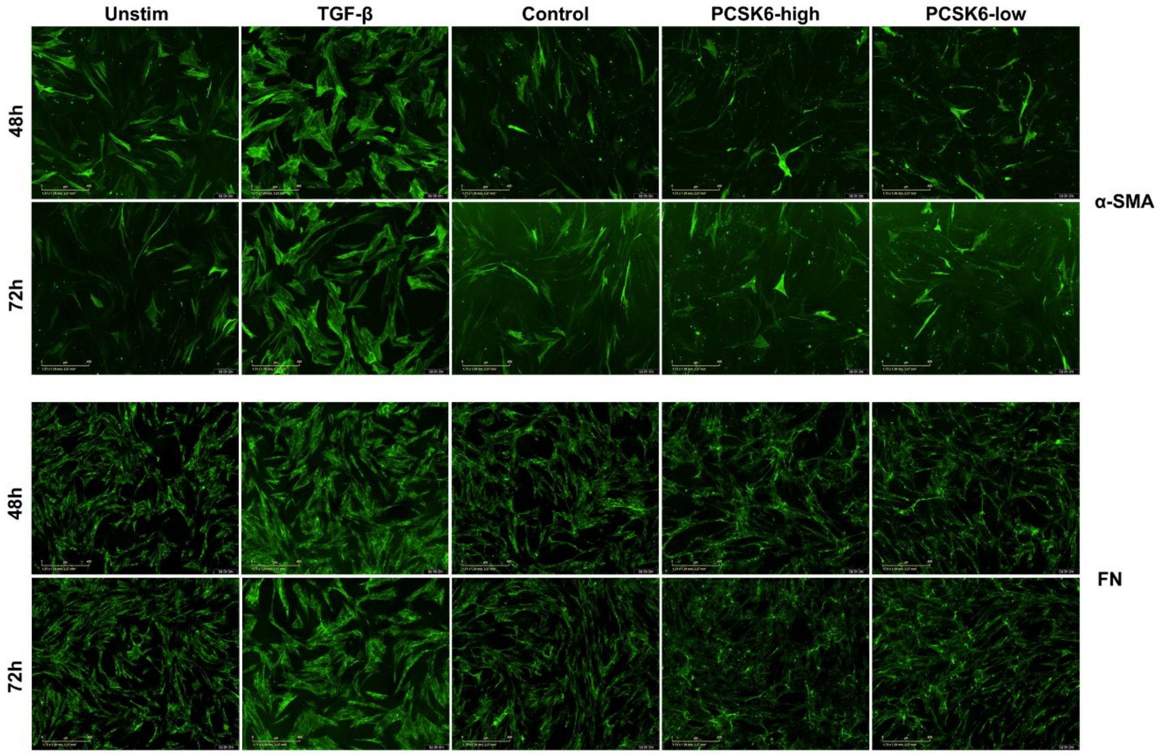

Figure S2. Representative images of immunofluorescence staining of α-SMA and fibronectin (FN) in NHLFs after 48, and 72 hours of stimulation with mCherry-control, PCSK6-high and PCSK6-low CM (*n*=3).

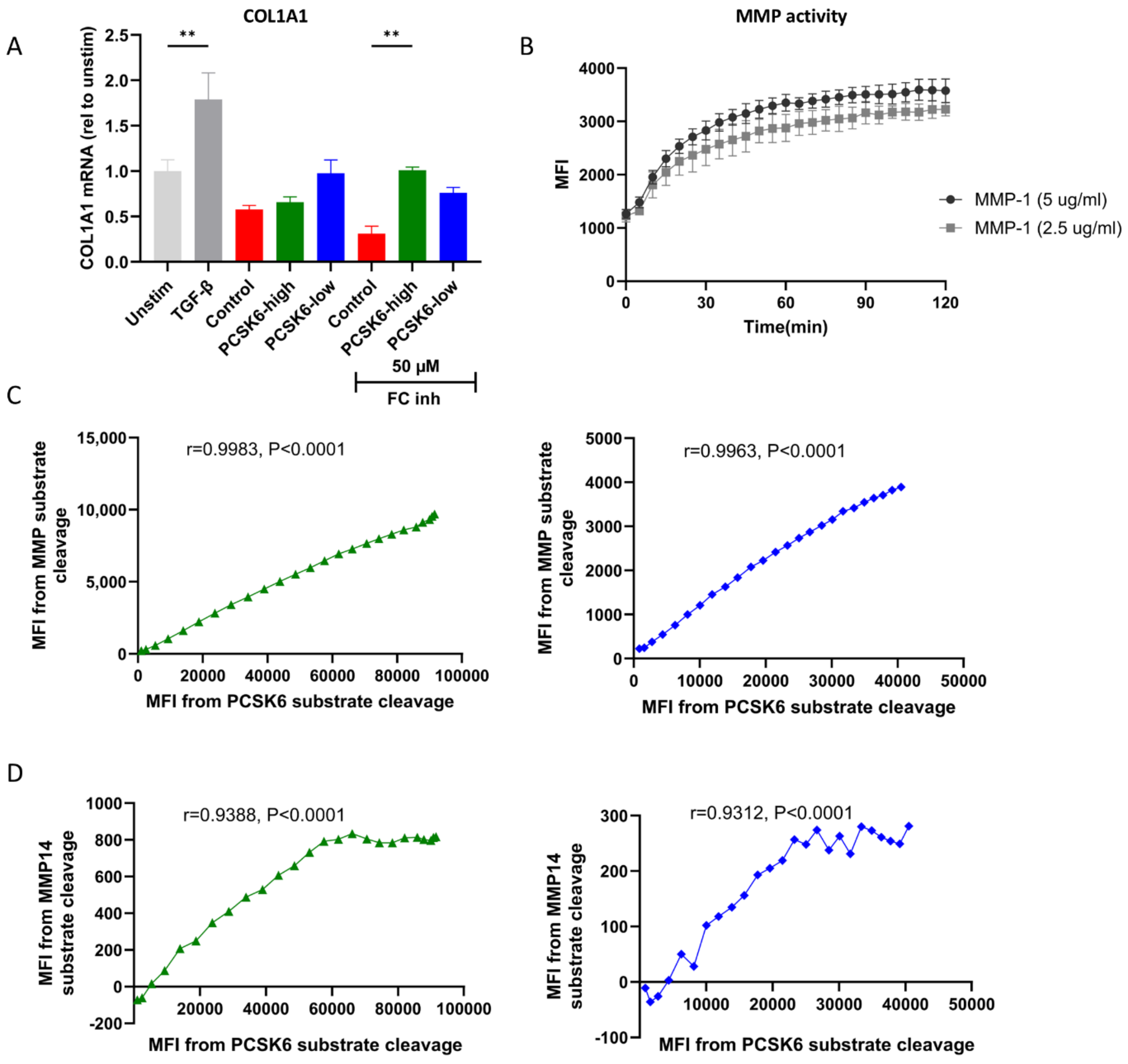

Figure S3. PCSK6 and MMP activity in CM is correlated. (A) Collagen type I mRNA expression levels in fibroblasts stimulated with CM from mCherry-control, GFP-high and GFP-low CM collected with or without the presence of 50 μM furin convertase (PCSK6) inhibitor (*n*=3). (B) Proteolytic activity of 2.5 and 5 μg/mL recombinant MMP-1 on the fluorogenic MMP substrate (*n*=3). (C) Correlation of proteolytic activity of PCSK6 and MMPs in CM from PCSK6-high (left panel) and PCSK6-low (right panel) as measured by PCSK6 and MMP substrate cleavage (*n*=3). (D) Correlation of proteolytic activity of PCSK6 and MMP14 in CM from PCSK6-high (left panel) and PCSK6-low (right panel) as measured by PCSK6 and MMP14 substrate cleavage (*n*=3). Fluorescence was measured at excitation 320/20 nm, emission 360/40 nm and normalized to the baseline fluorescence of the substrate peptide in the medium alone. MFI indicates mean channel fluorescence minus background. Data were expressed as mean ± SEM. Statistical significance was tested using one-way ANOVA. *p*-values: ** <0.01. Correlation analysis was computed with Pearson correlation.

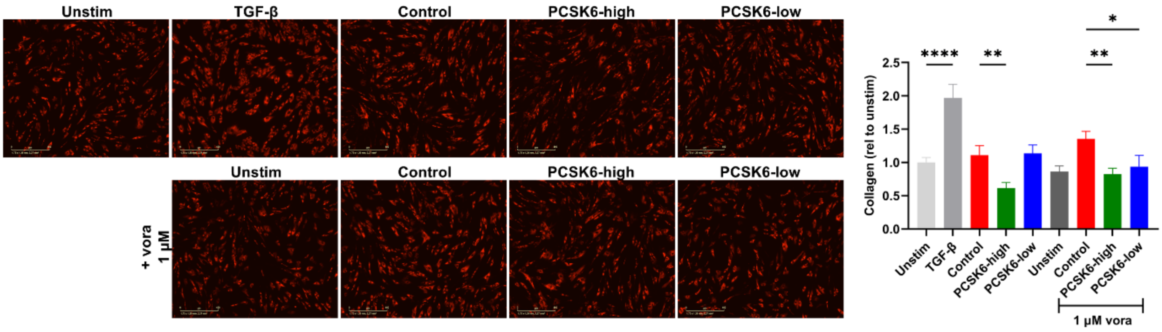

Figure S4. Inhibition of PAR-1 does not affect collagen deposition of fibroblasts exposed to mCherry-control, PCSK6-high or PCSK6-low CM. Representative images (10x magnification) of immunofluorescence staining for collagen type I in NHLFs 48 hours after stimulation with mCherry-control, PCSK6-high and PCSK6-low CM in the presence or absence of 1 µM PAR-1 inhibitor vorapaxar (vora). Vorapaxar was added to the NHLFs 1 hour prior to CM stimulation. Quantification of the fluorescent signal corrected for confluence is shown relative to unstimulated NHLFs. TGF-β stimulation served as a positive control (*n*=6). Scale bar = 400 µm. Results are expressed as mean ± SEM. Statistical significance was tested using one-way ANOVA. *p*-values: * <0.05, ** <0.01, **** <0.0001.

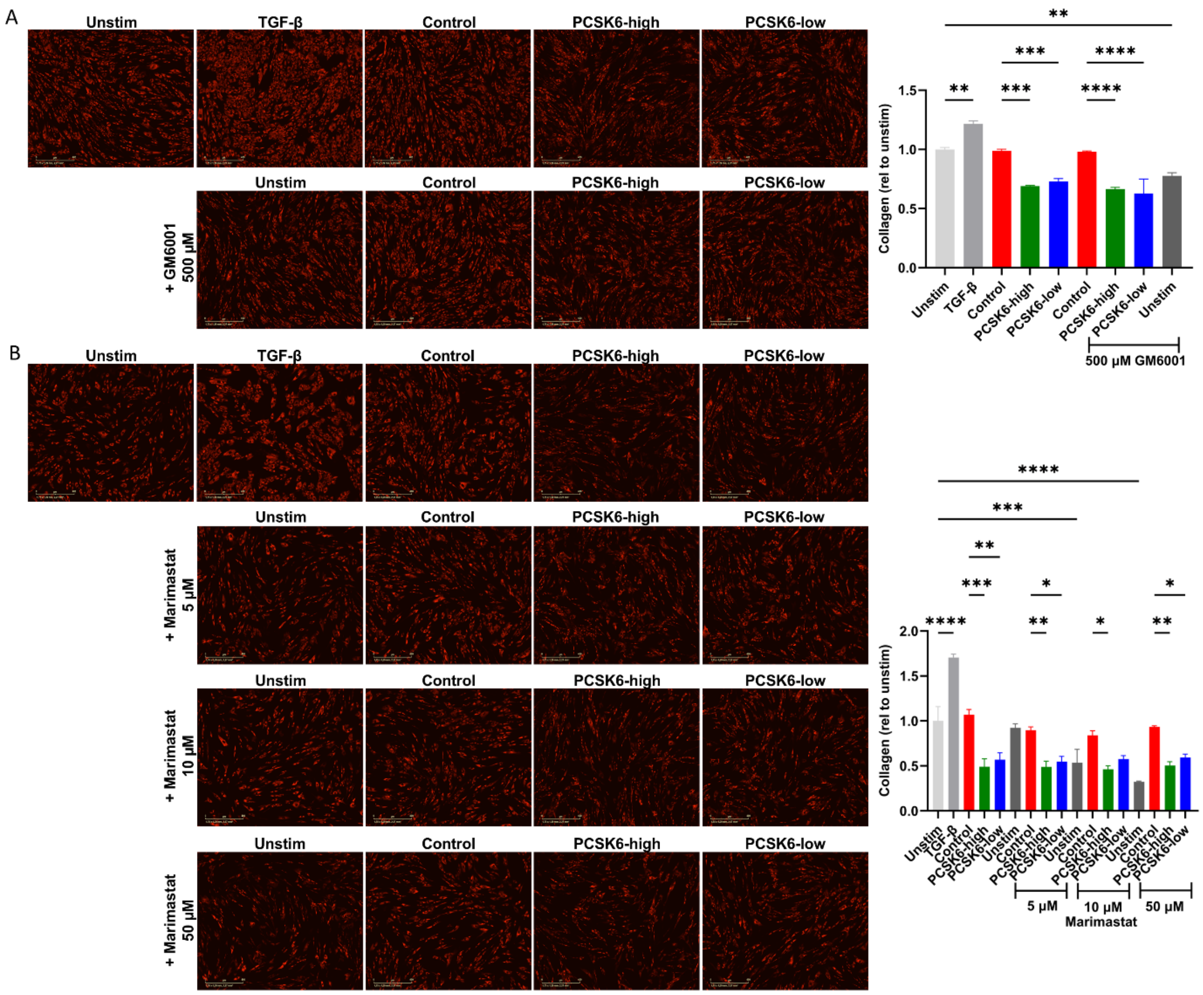

Figure S5. MMP inhibition using a single inhibitor does not reverse the decreased collagen levels induced by PCSK6 CM in fibroblasts. (A) Representative images (10x magnification) of immunofluorescence staining for collagen type I in NHLFs 48 hours after stimulation with mCherry-control, PCSK6-high and PCSK6-low CM in the presence or absence of 500 µM broadspectrum MMP inhibitor GM6001. GM6001 was added to CM prior to CM stimulation. Quantification of the fluorescent signal corrected for confluence is shown relative to unstimulated NHLFs. TGF-β stimulation served as a positive control (*n*=3). Scale bar = 400 µm. (B) Representative images (10x magnification) of immunofluorescence staining for collagen type I in NHLFs 48 hours after stimulation with mCherry-control, PCSK6-high and PCSK6-low CM in the presence or absence of 5 µM, 10 µM or 50 µM MMP inhibitor marimastat. Marimastat was added to CM prior to CM stimulation. Quantification of the fluorescent signal corrected for confluence is shown relative to unstimulated NHLFs. TGF-β stimulation served as a positive control (*n*=3). Scale bar = 400 µm. Results are expressed as mean ± SEM. Statistical significance was tested using one-way ANOVA. *p*-values: * <0.05, ** <0.01, *** <0.001 and **** <0.0001.
